# Characteristics and treatment outcomes of tuberculosis patients who “transfer-in” to health facilities in Harare City, Zimbabwe: a descriptive cross-sectional study

**DOI:** 10.1186/1471-2458-12-981

**Published:** 2012-11-15

**Authors:** Kudakwashe C Takarinda, Anthony D Harries, Tsitsi Mutasa-Apollo, Charles Sandy, Owen Mugurungi

**Affiliations:** 1 AIDS & TB Unit, Ministry of Health & Child Welfare, Harare, Zimbabwe; 2 Department of Community Medicine, University of Zimbabwe, Harare, Zimbabwe; 3 International Union Against Tuberculosis and Lung Disease, Paris, France; 4 London School of Hygiene and Tropical Medicine, London, UK

**Keywords:** Tuberculosis, Transfer-in, Treatment outcomes, Zimbabwe

## Abstract

**Background:**

Zimbabwe is among the 22 Tuberculosis (TB) high burden countries worldwide and runs a well-established, standardized recording and reporting system on case finding and treatment outcomes. During TB treatment, patients transfer-out and transfer-in to different health facilities, but there are few data from any national TB programmes about whether this process happens and if so to what extent. The aim of this study therefore was to describe the characteristics and outcomes of TB patients that transferred into Harare City health department clinics under the national TB programme. Specific objectives were to determine i) the proportion of a cohort of TB patients registered as transfer-in, ii) the characteristics and treatment outcomes of these transfer-in patients and iii) whether their treatment outcomes had been communicated back to their respective referral districts after completion of TB treatment.

**Methods:**

Data were abstracted from patient files and district TB registers for all transfer-in TB patients registered from January to December 2010 within Harare City. Descriptive statistics were calculated.

**Results:**

Of the 7,742 registered TB patients in 2010, 263 (3.5%) had transferred-in: 148 (56%) were males and overall median age was 33 years (IQR, 26–40). Most transfer-in patients (74%) came during the intensive phase of TB treatment, and 58% were from rural health-facilities. Of 176 patients with complete data on the time period between transfer-in and transfer-out, only 85 (48%) arrived for registration in Harare from referral districts within 1 week of being transferred-out. Transfer-in patients had 69% treatment success, but in 21% treatment outcome status was not evaluated. Overall, 3/212 (1.4%) transfer-in TB patients had their TB treatment outcomes reported back to their referral districts.

**Conclusion:**

There is need to devise better strategies of following up TB patients to their referral Directly Observed Treatment (DOT) centres from TB diagnosing centres to ensure that they arrive promptly and on time. Recording and reporting of information must improve and this can be done through training and supervision. Use of mobile phones and other technology to communicate TB treatment outcomes back to the referral districts would seem the obvious way to move forward on these issues.

## Background

Tuberculosis (TB) remains a disease of major public health concern the world over with most cases being found in the South-East Asia, African and Western Pacific regions [1]. Emergence of the human immunodeficiency virus (HIV) epidemic has fuelled the TB epidemic worldwide, with one in ten TB cases in 2010 being estimated to be HIV-positive [1]. The African region has been the worst affected by the HIV epidemic and accounts for over 80% of the global HIV-TB burden [1]. Zimbabwe has not been spared as it is one of the 22 World Health Organisation (WHO) high TB burden countries [1] and currently has an HIV prevalence of 15.2% for the adult population [2].

The World Health Organization has for nearly 20 years implemented a Stop TB Strategy for global and national TB control, and one of its cornerstones is a standardised recording and reporting system. For smear-positive pulmonary TB (PTB) patients, at the end of a course of treatment there are 6 possible outcomes: cure, treatment completed, failed, died, lost to follow up and transferred out [3,4]. A transfer-out is a patient who transfers from one reporting centre to another centre in a different reporting district and for whom the treatment outcome is unknown. This can be a sizeable problem: for the 2.5 million new smear-positive PTB patients registered in “DOTS” programmes globally in 2006, 3% or 75,000 patients were reported to be transferred out [5].

For the same year, the transfer-out rates in the African region were 4%, with individual countries reporting rates of between 0% – 16% [4]. From the two most recent global reports in 2010 and 2011[1,6], it is difficult to get a good estimate of the transfer-out problem as this treatment outcome category has been subsumed under a category called “not evaluated”: for the 2009 global cohort of treatment outcomes in new smear-positive PTB patients, the “not evaluated” proportion was 4% of 2.6 million patients registered [1], and this group probably contained a high number of patients who had transferred out.

Every patient who transfers out from the original registration unit should in theory “transfer-in” to a new TB registration unit in a different reporting district. However, the original registration unit maintains the responsibility for reporting on their treatment outcomes. Patients who are transferred into Harare City from another district carry with them a transfer out form from the referral district, which includes details such as patient name, referral hospital and district, sex, age, treatment category, type of TB and transfer-out date. These transfer-in patients are registered in the TB register at either of 2 infectious disease hospitals before referral for DOT at their nearest municipal clinic. Upon completion of TB treatment, the national guidelines specify that the outcomes of these transfer-in patients be routinely communicated back to the district from which the patient was transferred.

District TB coordinators for each district also follow up treatment outcomes of patients transferred out by issuing a treatment outcome request form to the transfer-in receiving health facility. If treatment outcomes of patients transferred out are unknown when cohort analysis is conducted, these patients have their treatment outcomes recorded as “transfer-out”. Including the treatment outcomes of patients transferred into a district as part of that district’s cohort is not allowed as this will result in more patients with treatment outcomes than patients notified and registered for that particular reporting period [3].

There are few data from national TB programmes about whether or to what extent this process happens. The aim of this study therefore was to describe the characteristics and outcomes of TB patients that transferred into Harare City health department clinics under the national TB programme. Specific objectives were to determine i) the proportion of a cohort of TB patients registered as transfer-in, ii) the characteristics and treatment outcomes of these transfer-in patients and iii) whether their treatment outcomes had been communicated back to their respective referral districts after completion of TB treatment.

## Methods

### Study design

This was a descriptive cross-sectional study design using registers and treatment cards.

### Study setting

This study was conducted in Harare, the capital city of Zimbabwe which has an estimated population of around 1.6 million [7]. In Harare, National TB programme services are offered under the city health department, and hence the study included all 32 municipal clinics and 2 infectious disease hospitals which offer general health services integrated with TB treatment services [7]. The municipal clinics are partitioned into either the southern or northern region, with each region consisting of an infectious disease hospital in which TB diagnostic services (smear microscopy and chest radiography) are performed for the surrounding clinics.

### General diagnosis and management of TB patients

In Zimbabwe, TB is diagnosed according to national guidelines [3] which are based on the WHO TB treatment guidelines [4]. Direct smear microscopy is the main method for diagnosing pulmonary TB, whereby suspected TB patients have their sputa collected at their nearest municipal clinic which are then sent for smear microscopy to the infectious disease hospital in their respective region. All confirmed TB patients are treated using standardised anti-TB regimens according to national [3] and international guidelines [4].

Monitoring is done clinically for smear-negative PTB and EPTB patients whilst those with new smear-positive PTB have sputum smears examined for acid-fast bacilli at 2, 5 and 6 months. Those with previously treated sputum smear-positive PTB have sputum examined at the end of 3,5 and 8 months. Smear-negative patients who complete treatment and smear-positive patients who complete treatment with or without negative smears are regarded as “successfully completing treatment”. Patients are offered HIV counselling and testing (opt-out provider-initiated) upon diagnosis of TB, and cotrimoxazole preventive therapy (CPT) is started together with anti-TB treatment for TB/HIV co-infected patients, provided there is no contra-indication. All HIV-positive TB patients are eligible for antiretroviral therapy (ART) initiation at Opportunistic Infections (OI) /ART initiating clinics at either of the 2 hospitals commencing patients on TB treatment in Harare.

### Patient sample

Data were collected for all TB patients that transferred into Harare City between 1 January 2010 and 31 December 2010 from TB registers at the two infectious disease hospitals.

### Data variables, data source and data collection

Patient data on those who transferred-in were abstracted from the TB registers between January 2012 and February 2012 using a data collection form, and variables that were collected included:- TB registration number, sex, age, type of TB, HIV status, TB treatment phase at time of transferring, date of transfer-out from the districts, date of transfer-in at the regional infectious disease hospital and the TB treatment outcome. Patient data in the TB registers were verified from individual patient files stored at their respective hospitals, and these patients were traced to their referral municipal clinics to establish if they arrived and were registered. District TB coordinators were also contacted by telephone to establish if treatment outcomes of their patients that had been transferred into Harare city had been communicated back to them. Individual TB registration numbers and respective dates of commencing TB treatment were used to trace these patients by district TB coordinators in their district TB registers.

### Statistical analysis

Patient information on the data collection forms was coded and single-entered electronically into Epidata version 3.1 (The Epidata Association, Odense Denmark). The data were then exported to Stata version 10 (Stata Corporation, College Station, Texas) for data cleaning and statistical analysis. Medians and inter-quartile ranges were calculated for skewed continuous variables whilst proportions were generated for categorical variables. Time between date of transfer out from the district and arrival at a health facility in Harare was calculated by subtracting the transfer out date written on the transfer-out slip from the TB registration date recorded in the TB register of the receiving infectious disease hospital in Harare City. Comparisons between proportions was done using the chi-square test or alternatively the Fischers Exact test and Odds ratios (OR) with their 95% confidence intervals. Levels of significance were set at 0.05.

### Ethics

Ethics approval was granted locally by the Medical Research Council of Zimbabwe and the International Union Against Tuberculosis and Lung Disease (The Union). Confidentiality of information drawn from the patient records was ensured by excluding the patient names during data collection whilst all data collection forms were kept in a safe and secure place accessible only to the investigator.

## Results

### Proportion of TB patients registered as transfer-in

There were 7,472 patients registered with TB, of whom 263 (3.5%) were recorded as transfer-in.

### Characteristics and treatment outcomes of transfer-in patients

Demographic and clinical characteristics of transfer-in patients in comparison to the non-transfer cohort are shown in Table 1. More of the patients who transferred into Harare City health department were males (N=148, 56%). The overall median age among these patients was 33 years (IQR, 26–40), with no differences between males and females, 34 years (IQR, 26–41) versus 30 years (IQR, 24–38), p=0.142 respectively. Most transfer-in patients, (N=222, 87%) had new TB, and of these 190 (88%) had pulmonary TB (PTB) of whom 67 (35%) patients were smear-positive. “Retreatment others” were the most common type of previously treated TB. There was a significantly greater proportion of previously treated patients among the transfers-in (13%) in comparison to non transfer-in patients (8.4%), p=0.016.

**Table 1:** Demographic and clinical characteristics of transfer-in vs. Non transfer-in patients TB patient in Harare City – 2010

Characteristic*			**Patient Group (n(%))**	**p-value**
		**Transfers-in (n=263)**	**Non Transfers-in (n=7029)**	
***Sex***	Male	148 (56))	3993 (55)	0.667
	Female	115 (44)	3276 (45)	
***Age group in years***	<15	21 (8)	777 (10.8)	0.025
***(n=260)***	15-25	36 (14)	657 (9.1)	
	25-44	160 (62)	4242 (58.8)	
	45-54	26 (10)	847 (11.7)	
	>54	17 (7)	686 (9.5)	
***Category of TB***	New	222 (87)	6603 (91.6)	0.016
***(n=256)***	Retreatment TB	34 (13)	606 (8.4)	
***Type of new TB***	Smear-positive PTB	67 (31)	1946 (29.5)	0.355
***(n=216)***	Smear-negative PTB	87 (40)	2949 (44.7)	
	EPTB	26 (12)	595 (9.0)	
	PTB smears not done	36 (17)	1113 (16.9)	
***Type of retreatment TB***	Relapse	10 (34)	196 (32.3)	>0.99
***(n=29)***	Retreatment other	17 (59)	351 (57.9)	
	Retreatment after default	1 (3)	20 (3.3)	
	Retreatment after failure	1 (3)	39 (6.4)	
***HIV test done***	Yes	232 (95)	4605 (63.9)	<0.01
***(n=243)***	No	11 (5)	2604 (36.1)	
***HIV status***	Positive	163 (71)	3847 (83.5)	<0.01
***(n=228)***	Negative	67 (29)	758 (16.5)	

*Variables in the table above have varying totals because missing data was excluded.

NB: Percentages may not always add up to 100 because of rounding off error.

HIV = human immunodeficiency virus; TB = tuberculosis; PTB = pulmonary TB, EPTB = extra-pulmonary TB.

Of the 232 (95%) patients in whom an HIV test had been performed, 163 (71%) were HIV-positive. Of these, 143 (89%) were documented to be on CPT, but only 38 (23%) were documented as accessing ART during TB treatment. In comparison to non transfer-in patients, a greater proportion of transfer-in patients were HIV tested (95% vs. 63.9%, p<0.01), however a lesser proportion were diagnosed HIV-positive; 71% vs. 84%, p<0.01.

Characteristics of the transfer process for transfer-in TB patients are shown in Table 2. Data were not available for a variable number of patients according to the field of enquiry. Of the 176 patients with data on time between transfer-out and transfer-in, only 85 (48%) transfer-in TB patients arrived in Harare within one week of transfer from their referral districts. The median time between transfer-out and transfer-in was 8 days (IQR, 4–20 days). At the time of transfer-out, 159 (74%) patients were in the intensive phase of TB treatment. The majority of patients, 123 (58%) were transferred-out from rural health facilities, of whom 88 (71%) were from mission (faith-based) hospitals. Two patients were commenced on TB treatment outside the country.

**Table 2:** Characteristics of the Transfer process of transfer-in TB patients in Harare City, Zimbabwe

Characteristic* (N=263)	**n (%)**
*Time period between transfer-out and transfer-in to hospitals in Harare City (n=176)*	
< 8 days	85 (48)
8 – 31 days	70 (40)
>31days	21 (12)
*Anti-TB treatment phase at time of transfer-in from the district (n=216)*	
Intensive phase	159 (74)
Continuation phase	57 (26)
*Type of referral health facility from where patients were transferred-out (n=212)*	
rural mission hospital	88 (42)
district hospital	52 (25)
urban municipal clinic	51 (25)
rural clinic	4 (2)
prison facility	5 (2)
private clinic	5 (2)
provincial hospital	5 (2)
outside Zimbabwe	2 (<1)
*Referral site location (n=212)*	
Urban	82 (39)
Rural	123 (58)
prison facility	5 (2)
outside Zimbabwe	2 (<1)
*Distance from Referral health facility to Harare City (km) (n=211)*	
≤40	45 (21)
41-150	69 (33)
151-300	66 (31)
≥300	83 (15)

NB: Percentages may not always add up to 100 because of the rounding off error.

*Variables in the table above have varying totals because missing data was excluded.

TB = tuberculosis; DOT = directly observed treatment.

**Figure 1:**
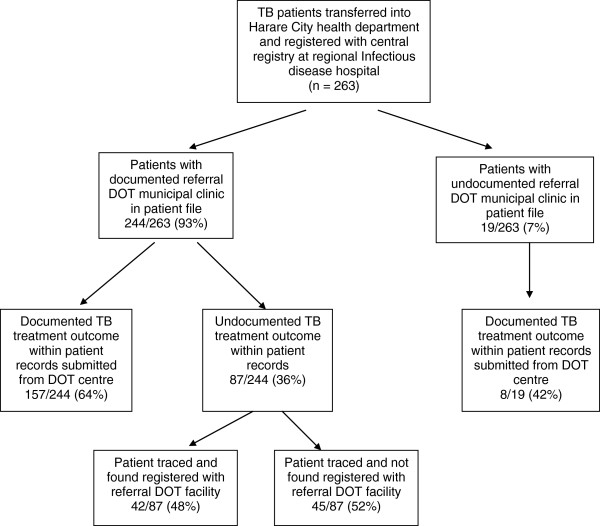
**Referral cascade of TB patients transferred into Harare City health department (Jan-Dec 2010).** NB: DOT = Directly Observed Treatment.

Figure 1 shows the referral cascade of transfer-in TB patients within Harare city. Patient files of all 263 transfer-in patients were taken from the records section at the infectious disease hospital in which they were registered. Of the 244 patients with documented referral to the DOT municipal clinic, 157 (64%) had their TB treatment outcomes recorded in the patient files. Of the 87 with missing treatment outcomes, a follow-up was conducted to the DOT centre to which they were referred, but only 42 (48%) were registered in the DOT registers.

Treatment outcomes of transfer-in TB patients and in relation to sex, age, type of TB, type of referral health facility and treatment phase of anti-TB treatment are shown in Table 3. Overall, 69% of patients successfully completed treatment, 10% had default/death/failure/or transfer-out again and in 21% of patients the treatment outcomes were not evaluated. Although there was some variation in treatment success in relation to characteristics of transfer-in patients, the only significant comparison was that better treatment success was experienced by new patients who transferred-in compared with retreatment patients (OR 2.6, 95% CI, 1.3 – 5.5, p < 0.01).

**Table 3:** Treatment outcomes of Transfer-in TB patients in relation to health facility and clinical characteristics

Characteristic* (N=263)	**TB treatment outcomes n (%)**						p-value**
	**Treatment success**	**Defaulters**	**Deaths**	**Failures**	**Transfers out**	**Missing outcomes**	
***Overall***	181 (69)	5 (2)	7 (3)	0 (0)	14 (5)	56 (21)	-
*Sex (n = 263)*							
Male	100 (68)	2 (1)	4 (3)	0 (0)	9 (6)	33 (22)	0.897
Female	81 (70)	3 (3)	3 (3)	0 (0)	5 (4)	23 (20)	
*Age group (n = 260)*							
<15	16 (76)	0 (0)	0 (0)	0 (0)	1 (5)	4 (19)	0.972
15-24	26 (72)	0 (0)	1 (3)	0 (0)	0 (0)	9 (25)	
25-44	108 (68)	4 (3)	5 (3)	0 (0)	10 (6)	33 (21)	
45-54	18 (69)	0 (0)	1 (4)	0 (0)	2 (8)	5 (19)	
≥55	11 (64)	1 (6)	0 (0)	0 (0)	1 (6)	4 (24)	
*Type of TB (n = 256)*							
New	161 (73)	4 (2)	5 (2)	0 (0)	9 (4)	43 (19)	0.026
retreatment	17 (50)	1 (3)	2 (6)	0 (0)	5 (15)	9 (26)	
*Time period between transferring from district and registration in Harare City (n = 176)*							
< 8 days	60 (71)	3 (4)	3 (4)	0 (0)	5 (6)	14 (16)	0.477
≥8 days	69 (76)	2 (2)	0 (0)	0 (0)	4 (4)	16 (18)	
*Type of referral health facility (n = 212)*							
rural mission hospital	62 (70)	2 (2)	2 (2)	0 (0)	4 (5)	18 (20)	0.355
district hospital	39 (75)	1 (2)	1 (2)	0 (0)	1 (2)	10 (19)	
municipal clinic	38 (75)	1 (2)	0 (0)	0 (0)	5 (10)	7 (14)	
rural clinic	2 (50)	0 (0)	0 (0)	0 (0)	2 (50)	0 (0)	
prison facility	2 (40)	0 (0)	1 (20)	0 (0)	0 (0)	2 (40)	
provincial hospital	4 (80)	0 (0)	1 (20)	0 (0)	0 (0)	0 (0)	
private clinic	2 (40)	0 (0)	0 (0)	0 (0)	0 (0)	3 (60)	
Outside Zimbabwe	1 (50)	0 (0)	0 (0)	0 (0)	0 (0)	1 (50)	
*Distance to referral health facility from Harare City (km) (n = 211)*							
≤ 40	27 (60)	1 (2)	1 (2)	0 (0)	5 (11)	11 (24)	0.179
41 – 150	51 (74)	2 (3)	1 (1)	0 (0)	0 (0)	15 (22)	
151 – 300	48 (73)	1 (2)	3 (5)	0 (0)	4 (6)	10 (15)	
>300	24 (77)	0 (0)	0 (0)	0 (0)	3 (10)	4 (13)	
*Treatment phase at time of transfer from referral health facility (n = 263)*							
initiation phase	112 (70)	5 (3)	3 (2)	0 (0)	11 (7)	28 (18)	0.053
continuation phase	42 (74)	0 (0)	0 (0)	0 (0)	1 (2)	14 (25)	
Missing	27 (57)	0 (0)	4 (9)	0 (0)	2 (4)	14 (30)	

NB: Percentages may not always add up to 100 because of rounding off error.

*Variables in the table above have varying totals because missing data was excluded.

** P-values are for the Fischers Exact test for associations between TB treatmement outcomes and clinical and health facility characteristics.

DOT = Directly Observed Treatment.

Treatment outcomes of transfer-in TB patients compared to those of the non transfer-in cohort are shown in Table 4. There was a lower proportion of treatment success among the transfer-in patients (69%) when compared to non transfer-in patients (83%) as there were more patients with missing treatment outcomes among the transfers-in (27% vs. 8%, p<0.01).

**Table 4:** Treatment outcomes of Transfer-in TB patients in comparison to the non Transfer-in cohort

**TB treatment outcome**		**Type of Patient n (%)**	p-value**
	**Transfers-in (n=263)**	Non Transfers-in* (n=7010)	
Cured	58 (22.1)	1769 (25.3)	0.581
Treatment completed	123 (46.8)	4007 (57.3)	0.020
Defaulters	5 (1.9)	105 (1.5)	0.943
Deaths	7 (2.7)	585 (8.4)	0.588
Treatment failure	0 (0)	19 (0.3)	-
Not evaluated	70 (26.6)	525 (7.5)	<0.01

NB: Percentages may not always add up to 100 because of rounding off error.

*TB treatment outcomes were reported for only 7,010 non transfer-in patients out of 7,029 patients notified in Harare City for 2010 as obtained from the Zimbabwe National TB Programme.

** P-values are for the Fischers Exact test for associations.

### Communication of treatment outcomes of transfer-in patients back to referring districts

There were 225 of the 263 patients documented as being transferred in from 42 referring health facilities. Of these, there were only 3 (1.3%) patients who had treatment outcomes notified back at the referring health facility. For these 3 patients, the distance between the transfer-out and transfer-in facility was between 41 and 150 Km, the referring facilities were all rural and the 3 patients had successfully completed treatment.

## Discussion

This study, the first of its kind in Zimbabwe, shows that although a small proportion of patients transferred-in to Harare city out of the total number of patients registered, the management according to guidelines was poor with only 1.3% of treatment outcomes being notified back to the referring unit. Transfer-in patients generally had new pulmonary TB, had largely been HIV-tested and had a high HIV-prevalence rate, not very different from the non transfer-in patients in Harare city and in general among patients in Zimbabwe that have previously been reported on [8,9].

Important lessons emerge from this study which can inform patient management. Most of the patients for whom there were data had transferred-in during the intensive phase of treatment, with 52% taking more than 1 week to transfer-out and transfer-in. For the 10% of patients in whom the transfer process takes longer, it is important that district TB officers ensure that the patients travel with an adequate supply of oral anti-TB drugs so that treatment is not interrupted. Although transfer-in retreatment patients were small in number, their management nevertheless is more complicated. The intensive phase of treatment necessitates intramuscular streptomycin for 2 months, and it would be generally prudent if these patients remained in their original treatment unit until the course of injections has been completed. Interruption of treatment is a potent cause of drug-resistance [10] and must be avoided at all costs.

Many patients transferred from long distances, often rural mission hospitals, and TB officers need to ensure that patients can afford their travel and are again well covered with the necessary drugs in case of travel delays or mishaps. Why such high numbers transfer-in from rural mission hospitals is not certain but may reflect many persons’ perception that church-related health services are of better quality than those run by government, and once patients feel better on anti-TB treatment they decide to return to their urban public health services for continuation of therapy [11].

Treatment outcomes of patients who transferred-in were rather similar to the non transfer-in cohort and to those reported from patients registered in their original units [8,9], although there was a high proportion of patients not evaluated and with missing outcomes. The reasons for this are unclear, but may relate to poor documentation in registers and treatment cards or death, lost-to-follow-up and further transfer-outs which are not reported to the health facilities. Whatever the reasons, this is unsatisfactory and needs correction.

Finally, the majority of transfer-in TB patient did not have their outcomes communicated to their referral districts. This means the referring units would report these patients as “transfer-out”, while they could report on true outcomes if only communication had occurred. The conventional means of communication has been in the past through submission of treatment outcome request forms by the referral districts to the receiving districts through the postal system. Reasons for not communicating TB treatment outcomes are unclear. However, anecdotal reports have often attributed this to a shortage of TB treatment outcome request forms coupled with poor perceptions of postal services in Zimbabwe by health workers which are perceived as inconvenient, unreliable and an outdated mode of communication. There is therefore a need to move with the times and for the TB Control Programme to consider new cheap and feasible communication strategies such as use of a toll-free number for use by health-workers to their mobile phones [12].

There has been very little previous published work on the issue of transfer-in and transfer-out in TB patients. One study in Malawi showed that it was very common for TB patients to transfer-out, but the procedures for transferring–in and matching the two sets of patients was very poor [13]. This is an area in need of improvement. The strengths of this study were that a large number of patients were evaluated and the work was done through the routine system.

Study limitations included the usual problem of completeness and accuracy of routinely recorded programme data and the fact that patients with unevaluated outcomes could not be traced to their respective physical addresses in order to establish their true outcomes. Comparison data on the non transfer-in patients were also obtained as aggregate data from national reports and could not be verified or authenticated.

## Conclusions

In conclusion, there is need to devise better strategies of following up TB patients to their referral DOT centres from TB diagnosing centres to ensure that they arrive promptly and on time with drugs supplies uninterrupted. There is a need to improve on recording and reporting of information and this can be done through training and supervision. Use of mobile phones and other technology to communicate TB treatment outcomes back to the referral districts would seem the obvious way to move forward on this issue.

## Competing interests

The authors declare that they have no competing interests.

## Authors’ contributions

KT designed the study, collected and analysed data, wrote the first draft and coordinated the writing of the subsequent drafts and the final paper. ADH, TA, CS, and OM contributed to the design of the study and review of all subsequent drafts of the paper. All authors read and approved the final paper.

## Pre-publication history

The pre-publication history for this paper can be accessed here:

http://www.biomedcentral.com/1471-2458/12/981/prepub
